# The Virtual Operative Assistant: An explainable artificial intelligence tool for simulation-based training in surgery and medicine

**DOI:** 10.1371/journal.pone.0229596

**Published:** 2020-02-27

**Authors:** Nykan Mirchi, Vincent Bissonnette, Recai Yilmaz, Nicole Ledwos, Alexander Winkler-Schwartz, Rolando F. Del Maestro

**Affiliations:** 1 Department of Neurology & Neurosurgery, Neurosurgical Simulation & Artificial Intelligence Learning Centre, Montreal Neurological Institute and Hospital, McGill University, Montreal, Quebec, Canada; 2 Division of Orthopaedic Surgery, Montreal General Hospital, McGill University, Montreal, Quebec, Canada; Politechnika Krakowska im Tadeusza Kosciuszki, POLAND

## Abstract

Simulation-based training is increasingly being used for assessment and training of psychomotor skills involved in medicine. The application of artificial intelligence and machine learning technologies has provided new methodologies to utilize large amounts of data for educational purposes. A significant criticism of the use of artificial intelligence in education has been a lack of transparency in the algorithms’ decision-making processes. This study aims to 1) introduce a new framework using explainable artificial intelligence for simulation-based training in surgery, and 2) validate the framework by creating the Virtual Operative Assistant, an automated educational feedback platform. Twenty-eight skilled participants (14 staff neurosurgeons, 4 fellows, 10 PGY 4–6 residents) and 22 novice participants (10 PGY 1–3 residents, 12 medical students) took part in this study. Participants performed a virtual reality subpial brain tumor resection task on the NeuroVR simulator using a simulated ultrasonic aspirator and bipolar. Metrics of performance were developed, and leave-one-out cross validation was employed to train and validate a support vector machine in Matlab. The classifier was combined with a unique educational system to build the Virtual Operative Assistant which provides users with automated feedback on their metric performance with regards to expert proficiency performance benchmarks. The Virtual Operative Assistant successfully classified skilled and novice participants using 4 metrics with an accuracy, specificity and sensitivity of 92, 82 and 100%, respectively. A 2-step feedback system was developed to provide participants with an immediate visual representation of their standing related to expert proficiency performance benchmarks. The educational system outlined establishes a basis for the potential role of integrating artificial intelligence and virtual reality simulation into surgical educational teaching. The potential of linking expertise classification, objective feedback based on proficiency benchmarks, and instructor input creates a novel educational tool by integrating these three components into a formative educational paradigm.

## Introduction

Advances in technology have allowed digital platforms to become integrated into educational programs. The use of these technologies allows automation of traditional forms of teaching while also re-defining valued educational goals.[1] Digital technologies can quantitate skill performance, which, when analysed with artificial intelligence can result in new perspectives on psychomotor expertise and its composites. This is particularly useful for understanding complex tasks that need to be deconstructed into small components to provide an appropriate scaffold for learning. While artificial intelligence methodologies have been employed to assess skill level on simulated tasks, efforts are needed to enhance the understanding of the classification mechanisms utilized.

The application of artificial intelligence (AI) and machine learning in various fields has substantially facilitated the evaluation of large and multivariate datasets.[2, 3] Several types of algorithms fit under the umbrella of machine learning. Recent literature and newer applications of AI are also focused on artificial neural networks and deep learning, subsets of AI inspired by the biological neural system.[4–7] Although these models have shown significant potential in economics, finance, and medical applications, a common criticism of deep learning is that its decision-making process is a “black box”.[6] Basically, this means that it is difficult to understand how an algorithm makes a particular decision. This is problematic in the context of education because transparency and trust are vital components of ensuring a successful connection between teacher and learner.[8] Transparency is also important for developing and implementing appropriate grading schemes and feedback mechanisms. Without such mechanisms, students report negative emotions, such as frustration and discomfort, when using technology for higher (post-secondary) education.[9] The development of a feedback system powered by AI, and based on transparency, addresses some of these issues.

The employment of AI methodology for identifying components of expertise lends itself well for the understanding and teaching of complex tasks.[8] Virtual reality surgical simulators generate large amounts of data from each individual’s specific operative performance.[5, 10, 11] This data can be analysed and distilled to quantitate performance and provide automated feedback to the operator. This not only provides an efficient way to understand expertise, but it can also uncover unique features of skill that may have gone previously unrecognized.[5, 11, 12] In this study, we outline an educational platform (The Virtual Operative Assistant) for complex technical task enhancement based on explainable machine learning, and validate this system with a complex neurosurgical skill. The development of an objective and automated feedback system would allow trainees to practice and perfect surgical skills before operating on patients.

Although a number of virtual reality surgical training simulator platforms such as the NeuroVR (previously known as the NeuroTouch)[13] and SimOrtho[14] have been developed, progress in the implementation of simulator feedback platforms has been problematic. In the context of surgery, visual rating scales such as the Objective Structured Assessment of Technical Skills (OSATS) tool are considered the gold standard for the assessment of simulated tasks.[15] While this method has been shown to be valid and reliable for some surgical tasks,[16] it relies on the presence of examiners, and is thus prone to subjectivity and it is highly resource dependent. This can be problematic for trainees as they are constantly relying on the presence of a limited number of surgical educators. Hence, there is a need for an automated and more objective method of providing feedback for simulation-based training. Our group has demonstrated that performance metrics from a virtual reality simulator correlate with current visual rating scales (like the OSATS), while offering additional advantages such as the assessment of force application which cannot be assessed visually.[17] Our group has also developed best practices guidelines for utilizing AI in surgical simulation studies based on systematic literature search.[18] The Virtual Operative Assistant outlined in this study offers objective and automated feedback for the learner based on performance metrics from virtual reality simulators, allowing for an enhanced understanding of the critical components of expert performance.

The framework presented here aims to 1) introduce a novel and flexible method for teaching in simulation-based training in surgery and medicine, 2) validate the method by developing the Virtual Operative Assistant, a virtual training platform for a complex neurosurgical procedure.

Our framework was successfully validated with a linear support vector machine (SVM) algorithm, capable of classifying two groups of different skill levels (skilled and novice) according to various metrics of surgical performance in a virtual reality brain tumor resection task.

We present our study by first, introducing data acquisition from a VR simulator, followed by the steps taken to create and select metrics of performance. A thorough explanation of the machine learning algorithm training and testing process is then provided. Finally, we discuss the steps to extract individual metric scores and present them to a new participant using the Virtual Operative Assistant.

## Methods

This study was approved by the McGill University Health Centre Research Ethics Board, Neurosciences-Psychiatry. All participants whose data was used to train the machine learning model signed an approved written consent form.

**Fig 1:**
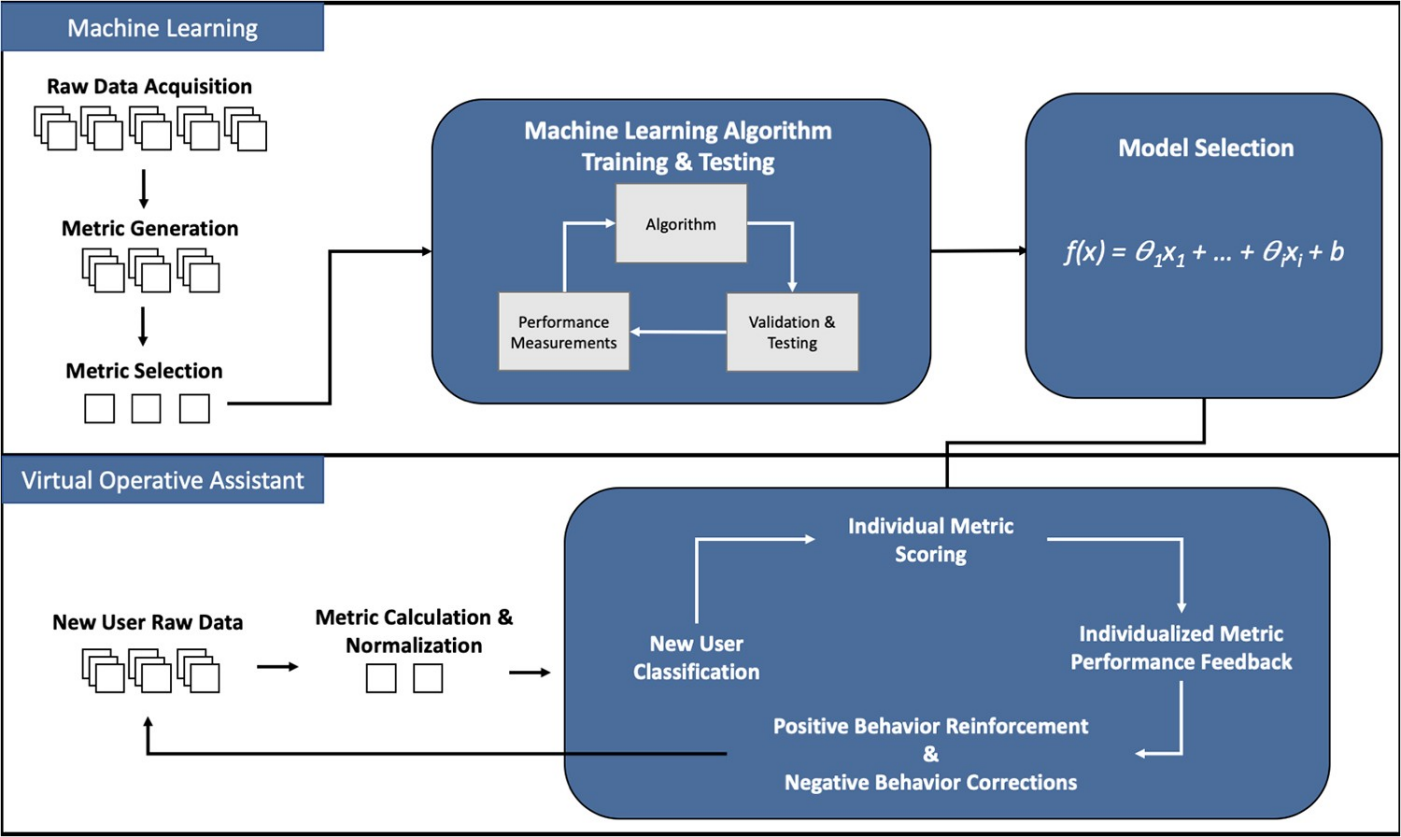
Framework for explainable artificial intelligence for medical simulation training. The top section provides an overview of standard machine learning methodology to obtain a predictive model. The bottom section harvests the power of the machine learning model for education. The simulation task generates raw data, which can be manipulated to create metrics of performance. Using algorithms, statistical methods, or expert opinions, metrics are selected based on their ability to differentiate between two or more groups. The selected metrics are fed to a machine learning algorithm for training and testing. A final predictive model can be selected based on predictive accuracy to build the Virtual Operative Assistant. Upon recruitment of new participants, their metrics of performance can be calculated and normalized. These are then fed to the Virtual Operative Assistant to provide a group classification (e.g. skilled or novice) as well as an individual breakdown of metric performance. The feedback reinforces positive behaviour while providing detailed information on which behaviours to improve. The Virtual Operative Assistant is an iterative program optimized for user learning.

Building an explainable machine learning powered teaching platform can be achieved following the framework in Fig 1. The system relies on a single processing unit known as the perceptron.[19] Building from this, the authors outline the rationale for employing the perceptron (also known as the linear Support Vector Machine) in this study in the S1 Appendix in S1 File.

### Raw data acquisition

The simulated medical or surgical task generates a large amount of data regarding how a user is interacting with the simulated scenario. This data can include basic measurements such as positioning of a surgical tool, and more complex measures such as tool rotation, forces applied on anatomical structures and volume of anatomical structures removed.

### Metric generation

The generation of performance metrics is accomplished utilizing three main methods. First, one can consult with experts in the field to create metrics representative of current measures of expertise. In the surgical field, for example, expert surgeons can be consulted to choose metrics that they believe reflect surgical performance in the surgical simulated task. Second, one can consult previous simulation studies and utilize these previously developed metrics. It is also useful to try to develop unique and previously unknown metrics able to differentiate expertise. In the surgical context, trainee instrument force application is difficult to assess by surgeon instructors. Computer platforms are capable of extracting both positional and quality components of trainee force utilization [20, 21] allowing quantification of these novel surgical performance metrics. This mechanism of metric generation may offer novel insight on the underlying constructs of expertise for the surgical scenario utilized. Efforts should be made to generate as many different metrics as possible, to try and capture the multiple facets of a given technical skill.

### Metric selection

Once metrics have been generated, the set of metrics must be narrowed to only those that are significant to be fed to the machine learning algorithm.[22] Several techniques can be used to achieve metric selection, although some of the most common include forward or backward feature selection. Forward feature selection involves an iterative process whereby metrics are added one by one and the algorithm’s predictive performance is tested for each iteration.[22] The final metric set is defined as the set where the highest accuracy is achieved. Backward feature selection starts with all the metrics and gradually removes one metric at a time while testing the algorithm’s performance.[22] The optimal set of metrics is defined by the point where the highest accuracy is reached. Since feature selection is an evolving research field, continually consulting the appropriate literature aids in selecting the optimal technique(s) for the simulation scenarios utilized.[23] This study employs a custom algorithm which combines forward and backward feature selection.[12]

### Machine learning algorithm training & testing

Once final metrics have been selected, they can be used to train supervised machine learning algorithms. The data should normalized to ensure all metrics are on the same scale. The dataset must be labelled (e.g. for medical simulation: expert/skilled or novice/less skilled) and contain a relatively large number of users. If the dataset is large enough, the labelled data should be split into two groups, one used for training and the other used for testing.[24, 25] The training set is used to train the algorithm to recognize different classes.[24, 25] An iterative cost reduction approach is employed during training,[26] whereby the algorithm alters values of the weights (*θ*) for each metric in order to reach the greatest predictive accuracy.

Once the model’s predictive power has been optimized, it is important to test the model on a new (previously unseen) dataset.[25] This step is important in order to detect overfitting of the model. Overfitting occurs when the model fits the training data too closely and cannot be generalized to new data with similar accuracy.[27] These models will perform poorly.

### Model selection

Once a model has been selected, it can be saved along with all its parameters. This model will be the core of the Virtual Operative Assistant.

### New user classification

New users can then be recruited to perform a task on the medical or surgical simulator. Following the previously described methods, raw data must be extracted from the simulator following completion of the simulated task. Using the same coded instructions that were previously used to generate metrics, the final metrics can be calculated for the new user. It is important to normalize the new data using the same methodology as the training data. A common normalization method is to calculate the z-score to ensure that all metrics are on the same relative scale.[28]

Once each metric has been normalized, they can be inputted into the Virtual Operative Assistant’s model. If the output of the model is positive, the new user belongs to class *Y* = +1. In the case of surgical simulation, this corresponds to a skilled participant. However, if the output of the model is negative, the new user would belong to class *Y* = -1. In surgical simulation, this corresponds to a novice participant. In addition, the probability of a user belonging to each class can be extracted to reflect expertise as a gradient rather than binary classes. This scoring method is more extensively explained in the S1 Appendix in S1 File.

### Individual metric scoring

The output of the Virtual Operative Assistant model relies on a series of inputs and corresponding weights. A unique advantage of the Virtual Operative Assistant is that it offers insight as to how each metric contributes to the model’s decision making. As outlined in the S1 Appendix in S1 File, a positive model output is optimized when each combination of metrics multiplied by their respective weight is also positive. Specific examples are discussed in the S2 Appendix in S1 File. This feature offers an in-depth insight into the algorithm to provide individualized performance feedback through the Virtual Operative Assistant.

### Framework validation

**Fig 2:**
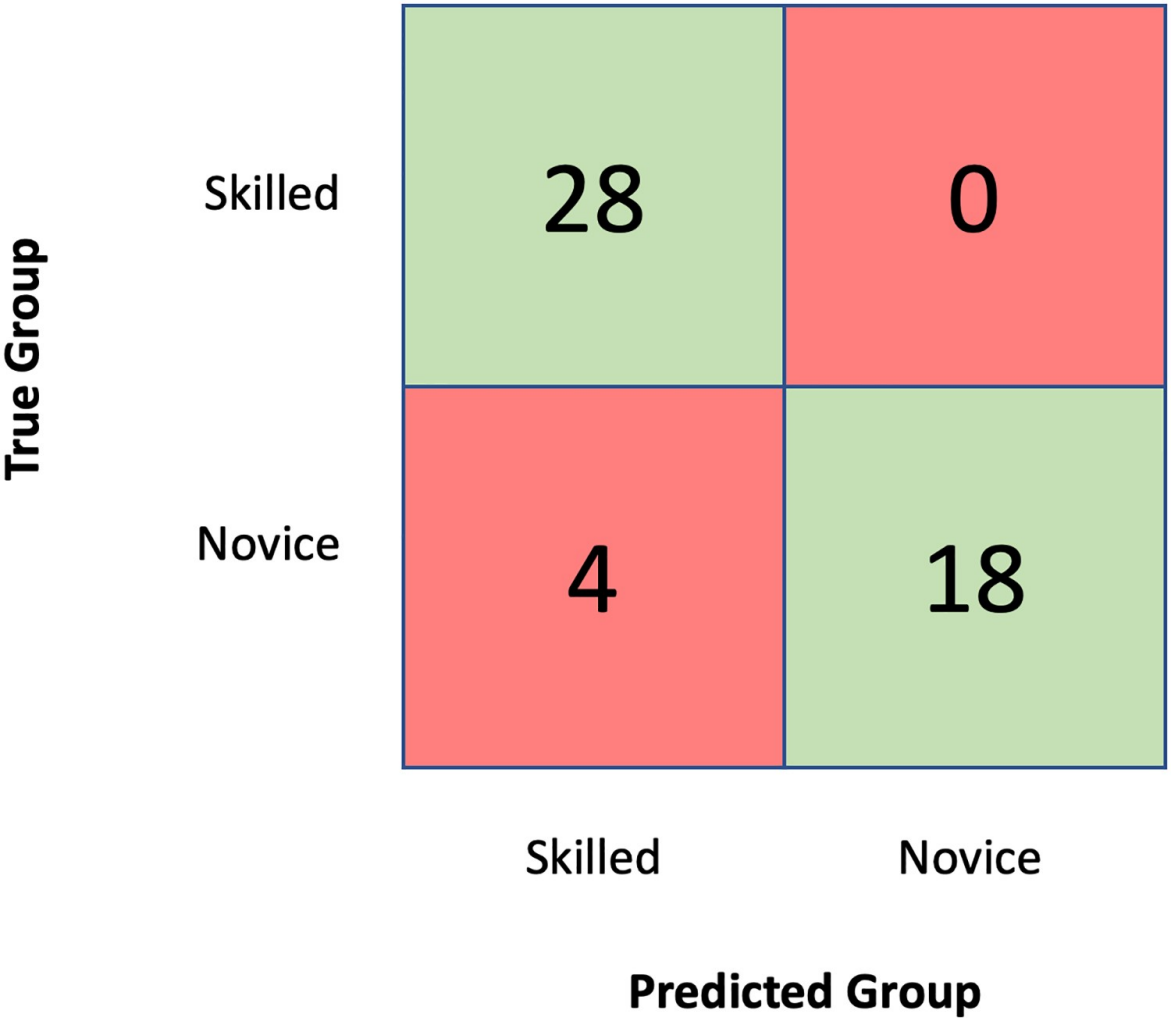
Confusion matrix for the classification of skilled and novice participants by the linear support vector machine. The algorithm correctly classified all skilled participants while correctly classifying 18 of the 22 novice participants (82%).

The model employed in this study to validate the Virtual Operative Assistant is a linear Support Vector Machine (SVM) developed in a previous study to evaluate a virtual reality brain tumor resection task on the NeuroVR simulation platform (CAE Healthcare, Montreal, Quebec, Canada).[12] The simulated scenario involves the removal of a subpial brain tumor using a simulated ultrasonic aspirator in the dominant hand, and a simulated bipolar in the non-dominant hand.[12] The task required the tumor to be removed completely while minimizing bleeding and healthy tissue damage. This model was originally developed to differentiate 4 groups of expertise. Since the present framework is designed for 2 groups the model was adapted to differentiate two groups, a skilled and a novice group. The participant demographics are described in Table 1. Fifty participants were recruited including 14 staff neurosurgeons, 4 neurosurgical fellows, 10 senior residents (postgraduate year 4 to 6), 10 junior residents (postgraduate year 1 to 3) and 12 medical students. The skilled group consisted of 28 participants including neurosurgeons, neurosurgical fellows and senior residents of postgraduate year 4 (PGY-4) and above. While the novice group consisted of 22 participants including junior residents PGY-3 and below and medical students. Two-hundred and seventy metrics of performance were created using the data recorded by the simulator.[12] Four final metrics were selected by using an iterative loop algorithm previously developed by our research group.[12] The metrics were normalized by calculating the z-scores and a support vector machine algorithm was trained using this data and the leave-one-out cross-validation accuracy was 92% (sensitivity: 100%; specificity: 82%). The confusion matrix in Fig 2 illustrates the classification results. No skilled participants were classified as novice and 4 novice participants were classified as skilled. The Virtual Operative Assistant was developed in Matlab R2018b. All code is publicly available at https://github.com/Ai-SimCenter/Virtual-Operative-Assistant/.

**Table 1:** Demographics information for two groups of participants performing the virtual reality neurosurgical task.

	Skilled (n = 28)	Novice (n = 22)
**Sex**		
**Male**	27	14
**Female**	1	8
**Level of Training**		
**Staff Neurosurgeons**	14	0
**Neurosurgical Fellows**	4	0
**Senior Residents (PGY4-6)**	10	0
**Junior Residents (PGY1-3)**	0	10
**Medical Students**	0	12

## Results

### Individualized metric performance feedback

The Virtual Operative Assistant was built and then validated with a complex virtual reality neurosurgical procedure involving the bimanual subpial removal of a lesion in the cerebral cortex which is commonly used in brain tumor and epilepsy surgery.[12] The S1 Video in the supplemental information demonstrates the simulated scenario. The system provides a metric-wise assessment of performance depending on the weight of each metric as described in the *Individual Metric Scoring* section above. If the weight is positive, the trainee should aim for a positive metric. Importantly, as these metrics are normalized through z-score calculation, this indicates that the trainee should aim to achieve a value higher than the mean of the training data to gain a positive normalized metric. If the weight is negative, the trainee should aim for a negative (or less positive) metric. Following the same logic, this corresponds to a value lower than the mean for the training data.

The neurosurgical model was composed of 4 metrics, each with a negative weight. These include 2 safety and 2 movement metrics as illustrated in Table 2. The weights provided information on the relative importance of each metric on the model’s decision-making process.[24, 29] A metric whose corresponding weight has a larger magnitude will play a greater role in the algorithm’s decision-making process.[29] Using this information, the metrics may be ranked to selectively train new users on the most important metrics first (briefly outlined in *Teaching with the Virtual Operative Assistant* section). Alternatively, the metric weights may simply be incorporated as part of a scoring system. Although the algorithm ranked the “Instrument tip distance” metric as most important, the authors opted to consult with experienced neurosurgeons to determine which metrics would be most important to teach first. Upon consultation, the safety metrics were deemed to be more important than movement metrics. This was done to ensure that the Virtual Operative Assistant resembled the current focus of neurosurgical education.[30]

**Table 2:** Selected metrics of performance for simulated neurosurgical task.

Category	Label	Description	Weight
**Safety**	Max Force w/ Bipolar	Maximum force applied by the user while using the bipolar instrument in their non-dominant hand.	-0.6002
	Rate of Bleeding	Rate of bleeding of the simulated patient.	-0.5106
**Movement**	Instrument Tip Distance	Mean distance between tip of the bipolar and ultrasonic aspirator instruments.	-1.4902
	Acceleration w/ Bipolar	Mean acceleration of the bipolar instrument.	-0.2710

### Teaching with the Virtual Operative Assistant

**Fig 3:**
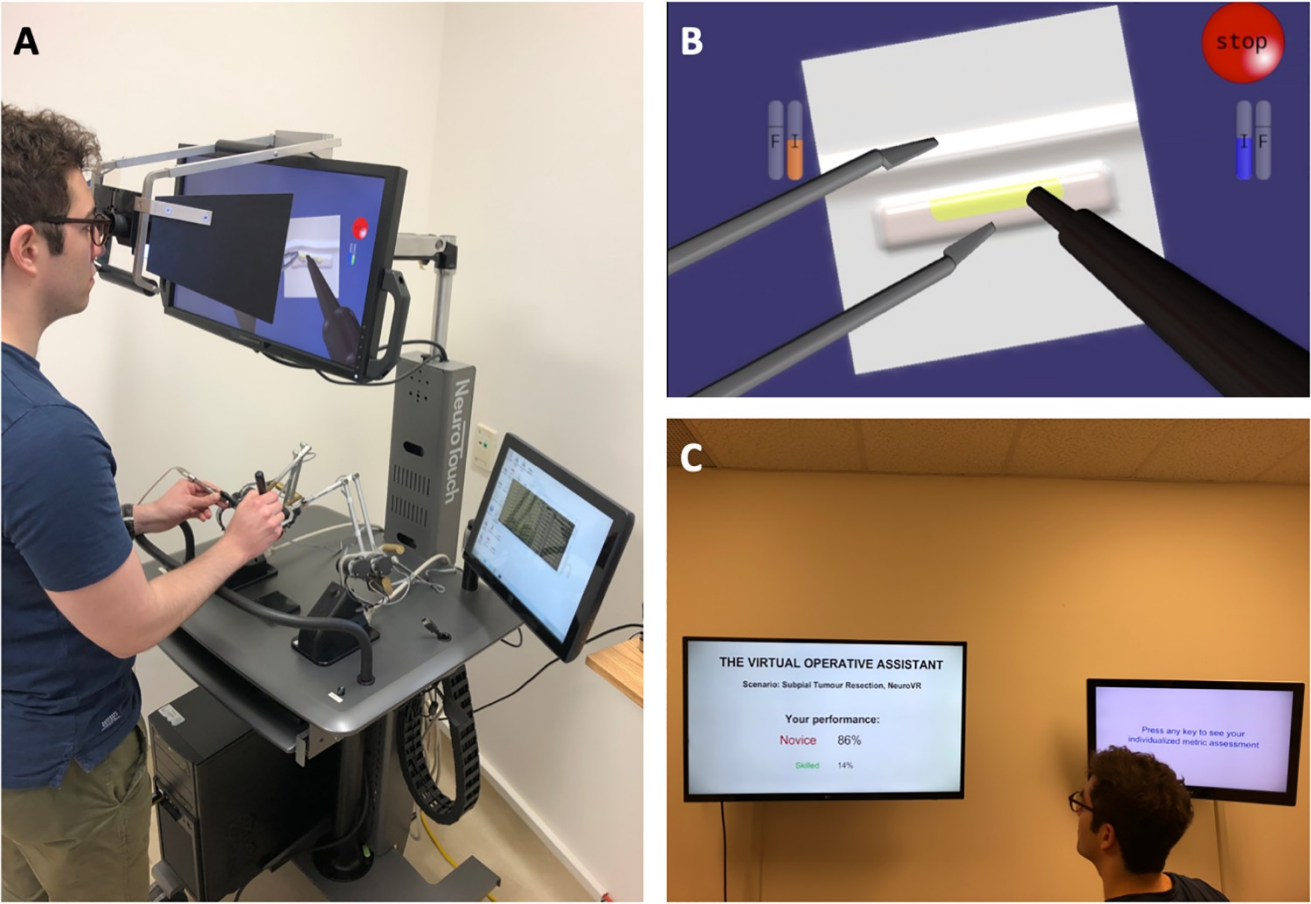
Educational paradigm of the Virtual Operative Assistant. (A) The trainee performs a simulated subpial tumor resection scenario on the NeuroVR (CAE Healthcare, Montreal, Quebec, Canada) platform using a simulated ultrasonic aspirator in the trainee’s dominant hand and a simulated bipolar in the non-dominant hand. (B) The scenario involves removal of a cortical tumor (yellow) with minimal damage to healthy brain regions (white). (C) Upon completion of the simulated task, the data is automatically saved and uploaded to the Virtual Operative Assistant software to provide instant feedback on two monitors.

To demonstrate the teaching potential of the framework, a teaching paradigm for a simulated neurosurgical scenario is illustrated in Fig 3.

**Fig 4:**
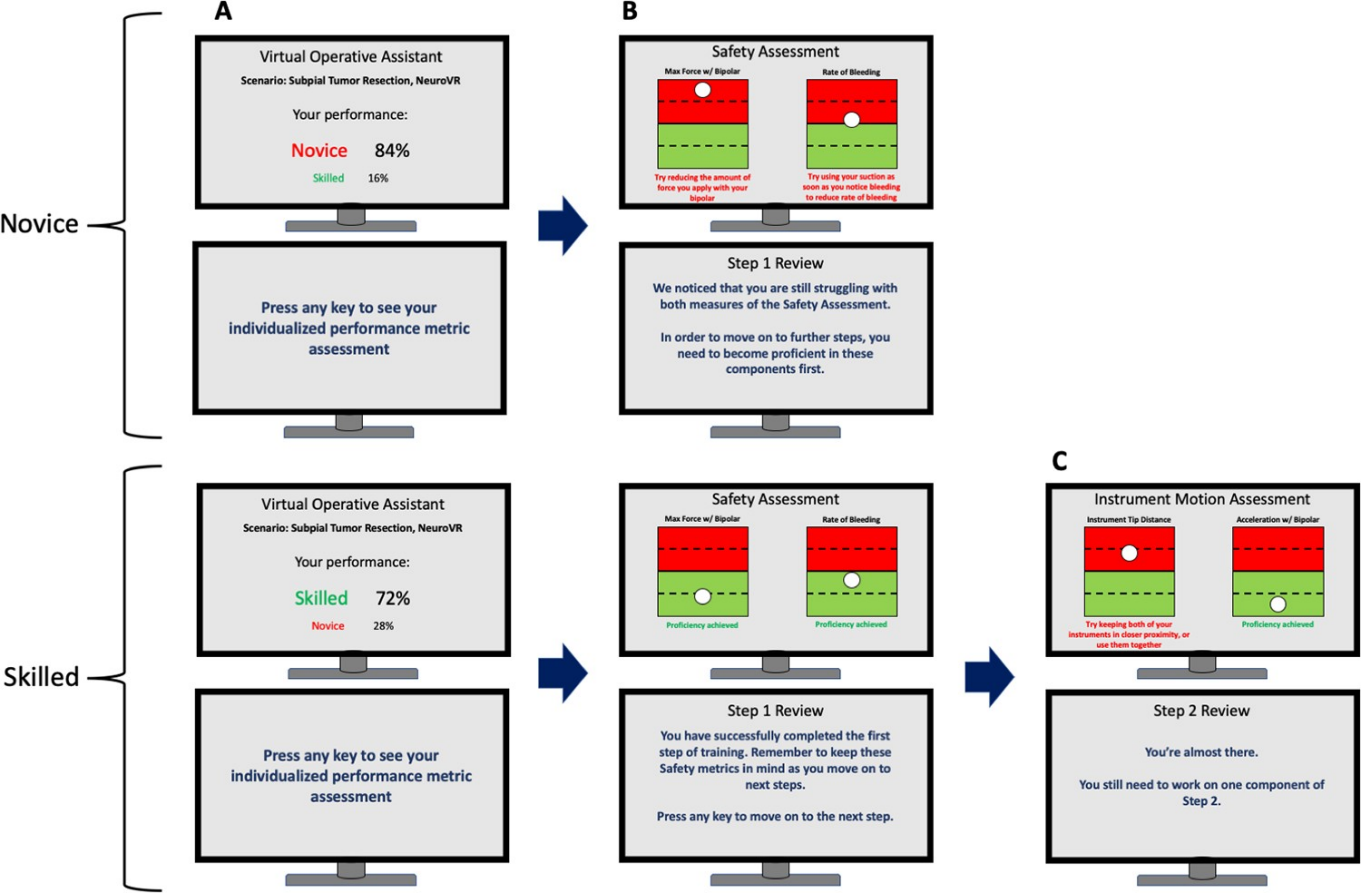
Performance assessment with the Virtual Operative Assistant. User received feedback on performance on two monitors. (A) The first screen informs the trainee of their classification along with a corresponding percentage for each class. (B) The second screen provides individualized feedback on 2 safety metrics. Metrics are accompanied with a positive statement (if competency achieved) or instructions to improve (if failed to achieve competency). Overall written and auditory feedback is provided on the second monitor. If the trainee has not achieved competency in all safety metrics (top row), the trainee cannot proceed further and must redo the scenario. If the trainee has achieved competency in all safety metrics (bottom row), the trainee can move on to step 2. (C) The third screen provides individualized feedback on 2 instrument motion metrics, accompanied by statements on their performance.

Following the principles of cognitive load theory,[31] the Virtual Operative Assistant aims to limit the amount of information immediately provided to the user to facilitate learning. This is achieved by categorizing the metrics into groups, a process also known as chunking,[32] while providing feedback in a stepwise manner. This approach can be visualised in Fig 4.

Upon completion of the simulated task, the user receives feedback on two monitors, divided into two steps, each containing two metrics. The machine learning algorithm initially computes a classification as “skilled” or “novice”. As expertise is often regarded as a gradient rather than a binary classification, the user’s classification is presented as a percentage of each class. This was followed by a metric-wise assessment. The first step includes metrics related to safety, while the second step contains metrics related to movements of the simulated instruments. Red zones indicate a novice performance for the respective metric, whereas green zones indicate a skilled performance. The boundary between the two zones is defined as the mean of all training data (z-score of 0). The paradigm is iterative, where the user must achieve full competency in all metrics of Step 1, in order to move on to Step 2. If the user fails to do so, this individual must repeat the task until competency is achieved. This follows the mastery learning model[33] by encouraging the trainee to master a component of the task before moving on. As highlighted by Block and Burns,[33] this technique ensures that the trainee has relevant skills to make subsequent steps of training more achievable, while imposing a realistic challenge for the trainee. A summary of the user’s performance is also provided at every iteration below each metric. The feedback reinforces the user’s positive behaviours where competency was achieved, while also addressing the metrics where the user performed less well. The summary incorporates automated auditory and video-based instruction specific to the psychomotor metric issues identified. This feature is designed to mimic current operating room surgeon instructor feedback strategies while allowing for self-guided learning. The text feedback provides a breakdown of positive behaviours and behaviours to improve. The auditory components provide more holistic feedback imitating what the learner would receive from the surgeon instructor in the operating room. The video-based feedback allows the trainee to compare their performance to that of skilled operator. We recognize that some individual psychomotor metrics may be difficult to teach and for the trainee to learn without special instruction. In the operating room a skilled surgeon instructor would first perform the complex task carefully outlining the critical steps to the learner. To model this component of operating room instruction the Virtual Operative Assistant incorporates a video for each metric illustrating how a skilled surgeon would perform the respective metric.

## Discussion

In this study, we investigated a transparency framework for machine learning to create an educational platform for complex psychomotor tasks. We achieved both aims of the study by 1) successfully introducing the steps of a novel teaching framework for simulation-based training, and 2) successfully validating our framework with a complex neurosurgical task through the Virtual Operative Assistant. We relied on a strong fidelity between the educational platform (Virtual Operative Assistant) and how the machine learning algorithm computes a trainee’s performance in a task.

### Transparency in education

Digital platforms have become increasingly common components of educational paradigms.[34] However, research involving the use of technology in higher education has identified neglect, frustration, uncertainty, need for confirmation and discomfort to be the primary negative emotions experienced by students.[9] These issues are believed to be due to “lack of feedback or faulty interactions”.[9] The concept of transparency in grading is central to addressing these issues. The transparency framework developed in this study directly addresses these challenges.

### Benefits of the Virtual Operative Assistant

The Virtual Operative Assistant offers many advantages over non-AI methods of teaching in medical simulation. It allows educators to identify individual components of psychomotor expertise in tasks too multifaceted for instructors to adequately appreciate without this technology. The complexity of surgery makes it an ideal educational paradigm to apply these systems.[35] Teaching in surgery is largely reliant on the apprenticeship model whereby the knowledge of a skill (know-how) is assumed to be learned through observation and graduated active responsibility in the operating room.[36] Therefore, a significant part of knowledge and skill acquisition also occurs through experiential learning. Trainees are often expected to learn a combination of complex skills to achieve expertise, without a comprehensive understanding of the individual components of holistic surgical competency. This form of training allows non-experts to automatically perform a given task after a number of repetitions, but without the inherent ability to shift between the automatic state and a more effortful state (i.e. a state where every motion is the result of a thinking process) capable of dealing with complications. This is analogous to driving, where most experienced drivers are experts and drive their car in an effortless automatic manner. A sudden unexpected event such as losing control of the vehicle on ice forces the experienced driver to dissociate from this automatic state to a more effortful state to deal with unexpected events. Moulton et al. discuss the importance of decoding the automatic as an essence to become an expert in a task.[37] The framework presented in this study allows medical educators to gain insight into the significant components that make up their skills. The Virtual Operative Assistant not only reveals significant metrics, but also incorporates the relationship between different metrics rather than assessing each metric individually. This allows trainees to understand how good performance in one metric may be compensating for a poor performance in another. The Virtual Operative Assistant outlines this issue and guides trainees along a path to achieve competency in all critical metrics. The Virtual Operative Assistant is designed to mimic real life training based on the well-established apprenticeship model employed to train surgeons and medical trainees. The system provides a similar feedback model as currently utilized by expert instructors to less skilled learners in the clinical or operating environment. Auditory feedback with a human voice reinforces components of the task that were correctly done and explains other components that require improvements. The video-based feedback demonstrates and reinforces the technical psychomotor skills necessary for attaining successful competence in each metric. This mimics real life experience where the surgeon will perform and thus outline to the learner the actual bimanual psychomotor skills necessary for safe operative outcomes. The Virtual Operative Assistant is an objective assessment tool which allows for self-guided and standardized medical or surgical training amongst learners. Studies have utilized machine learning to assess 2D visual images of operative procedures[38] and neuroanatomy.[39] However these systems do not allow for an assessment of quantitative instrument force application and instrument movements in a 3D operative environment nor provide verbal, visual and auditory feedback which may enhance the learning opportunities for the trainee.[10, 21, 40]

### Flexibility of the transparency framework

The design and presentation of information are vital components of digital educational platforms to optimize learning. Norman describes design in three different levels: visceral, behavioural, and reflective.[41] When applied to the Virtual Operative Assistant, visceral refers to the look of the platform, behavioural refers to the manner by which users and computers interact with each other, and reflective involves impression and judgement. The Virtual Operative Assistant addresses the behavioural level by providing feedback, and the reflective level by mimicking familiar feedback through audition and visual components.[9, 42] Studies across a variety of fields have proven the benefit of feedback to enhance skill acquisition and overall performance.[43] The framework is capable of adapting to the best practices of adult learning theories. As educational theories evolve, the information generated by the framework may be recombined in new ways taking advantage of systems available in the outlined Virtual Operative Assistant. The transparency framework presented places no restrictions on the platform interface. We present one type of interface in our Virtual Operative Assistant adapted to a neurosurgical scenario, however, the transparency framework is designed to allow new users to present the information in any way they like. This principle allows for flexibility to meet the visceral level of design. Although we present the transparency framework as applied to virtual reality surgical simulation, it can be employed to teach any form of complex task where a multivariate dataset can be obtained from experts and non-experts.

### Limitations

Limitations exist when utilizing the AI-powered feedback platforms. First, the framework was developed with a Support Vector Machines (SVM) algorithm distinguishing between two groups of expertise. Machine learning algorithms have the potential to distinguish between more than two groups of expertise.[12, 44] Studies are presently ongoing to adapt the framework to multi-layered perceptron such as artificial neural networks and to modify the framework for multiclass classification.[5] Second, the machine learning model is composed of metrics, weights, and a bias (extensively discussion in the S1 Appendix in S1 File). The bias is a weight without a corresponding metric.[45] Hence although the bias alters the decision boundary of the Virtual Operative Assistant, it is left out of metric-wise assessment. Third, another limitation of linear machine learning algorithms is that a very high positive score in one metric may overcompensate for other smaller negative metrics thereby impacting the classification. The authors believe that the Virtual Operative Assistant’s individualized metric feedback offers the necessary transparency for users to identify these overcompensation situations. The system also avoids these cases through the step-wise teaching methods where competency in all metrics is necessary for success rather than competency in just a few metrics. Although video feedback is useful, it can be challenging for future users of the framework to record a video illustrating expertise in a metric as there may be multiple different ways that expertise in a metric could be achieved. To deal with this issue we are developing a system that can record the entire simulation and automatically extract the time point(s) and event(s) that led the algorithm to classify the user performance as novice for a particular metric. This shortened clip would be presented to the user along with film footage of expert performance to allow for self-reflection and direct comparison between skilled and novice execution of that defined metric.

### Social and ethical impacts of AI-based teaching

It is important to recognize the potential social and ethical impacts of integrating artificial intelligence into educational paradigms. Medicine, like other disciplines, is influenced by developing trends in other scientific areas. Algorithms will need to be updated to reflect both the evolution of present AI methodologies and development of new medical technologies. The optimal frequency of algorithm updating is unknown. Frequent updating may allow insignificant time to verify an algorism’s teaching potential and limitations, while rare updates may cause the algorithm’s perception of expertise to fall behind the evolving definition.

Another important consideration of AI-powered teaching is whether the platform allows users to train to become more skilled or simply lays out a path for users to understand how to “cheat” the algorithm and give the illusion of being skilled. It is important to recognize that AI-based teaching platforms should undergo rigorous validation through experts’ opinion and multi-institutional studies to assess transferability of expertise to real life scenarios.

AI-based teaching platforms suffer from their inability to replicate the affective component of feedback. These systems struggle to understand the current emotional and cognitive state of the learner and cannot always tailor feedback in a contextually appropriate manner. Research indicates that feedback can be ineffective or occasionally detrimental to skill acquisition if the learner feels disconnected from the instructor or the feedback is not disseminated appropriately.[46] While automated feedback may provide more objective and standardized training, human-to-human connection is also an important component of educational paradigms. AI-based teaching platforms should be integrated into multifactorial educational systems that are carefully constructed and evaluated utilizing a best practices approach. These methodologies should be incorporated with the optimal participation of human instructors in the learning process.

### Conclusions and future directions

The Virtual Operative Assistant outlined in this study establishes a basis for the potential role of integrating artificial intelligence and virtual reality simulation into surgical and medical educational teaching. We demonstrate the ability of machine learning algorithms to not only different levels of expertise, but also identify teachable metrics which contribute to this classification. Our initial validation with a linear SVM revealed the feasibility of using the framework to build an automated and objective feedback platform for a complex neurosurgical task. The potential of linking expertise classification, objective feedback based on proficiency benchmarks, and instructor input creates a novel educational tool by integrating these three components into a formative educational paradigm.

Future work will aim to employ an explainable approach with other algorithms including artificial neural networks and deep learning to assess surgical performance. Recent studies by our group have shown the ability of artificial neural networks to differentiate 3 groups of expertise level performing a spine surgery procedure in virtual reality.[5] As such we aim to adapt our framework to be able to provide feedback to more than 2 groups of expertise. We are also performing trials to determine both the optimal method of presenting the complex information derived from the Virtual Operative Assistant to the trainee, and to determine if expert instructors working with the Virtual Operative Assistant improves performance. We have developed a single-blinded 2 arm randomized controlled trial employing an AI focused surgical training curriculum in which half the surgical trainees will be trained, and the other half will act as controls. Their performance will be assessed in live operative settings to obtain predictive validation of the AI systems utilized. These studies will help establish the future role of AI assisted learning in surgical education.

## Supporting information

S1 File (DOCX)

S1 Fig Brief outline of algorithms learning based on training data. (TIFF)

S2 Fig Sigmoid function representing the relationship between hypothesis output and probability of correct classification. (TIF)

S1 Video Demonstration of the virtual reality subpial simulation scenario. The participant holds a simulated ultrasonic aspirator in their dominant hand and a simulated bipolar in their non-dominant hand. The ultrasonic aspirator is used to remove the simulated tumor (yellow region) while the bipolar can be used to hold surrounding tissue (white regions) or coagulate bleeding.
(MOV)

## References

[1] McArthur D, Lewis M, Bishary M. The roles of artificial intelligence in education: current progress and future prospects. Journal of Educational Technology. 2005;1(4):42–80.

[2] McCarthy J, Minsky ML, Rochester N, Shannon CE. A proposal for the dartmouth summer research project on artificial intelligence, august 31, 1955. AI magazine. 2006;27(4):12.

[3] Kotsiantis SB, Zaharakis I, Pintelas P. Supervised machine learning: A review of classification techniques. Emerging artificial intelligence applications in computer engineering. 2007;160:3–24.

[4] Schmidhuber J. Deep learning in neural networks: An overview. Neural networks. 2015;61:85–117. 10.1016/j.neunet.2014.09.003 25462637

[5] Mirchi N, Bissonnette V, Ledwos N, Winkler-Schwartz A, Yilmaz R, Karlik B, et al Artificial Neural Networks to Assess Virtual Reality Anterior Cervical Discectomy Performance. Operative Neurosurgery. 2019 . 31832652 10.1093/ons/opz359

[6] Grapov D, Fahrmann J, Wanichthanarak K, Khoomrung S. Rise of deep learning for genomic, proteomic, and metabolomic data integration in precision medicine. Omics: a journal of integrative biology. 2018;22(10):630–6. 10.1089/omi.2018.0097 30124358 PMC6207407

[7] Esteva A, Robicquet A, Ramsundar B, Kuleshov V, DePristo M, Chou K, et al A guide to deep learning in healthcare. Nature medicine. 2019;25(1):24–9. 10.1038/s41591-018-0316-z 30617335

[8] Conati C, Porayska-Pomsta K, Mavrikis M. AI in Education needs interpretable machine learning: Lessons from Open Learner Modelling. arXiv preprint arXiv:180700154. 2018.

[9] Saplacan D, Herstad J, Pajalic Z. Feedback from Digital Systems Used in Higher Education: An Inquiry into Triggered Emotions-Two Universal Design Oriented Solutions for a Better User Experience. Studies in health technology and informatics. 2018;256:421–30. 30371503

[10] Sawaya R, Alsideiri G, Bugdadi A, Winkler-Schwartz A, Azarnoush H, Bajunaid K, et al Development of a performance model for virtual reality tumor resections. Journal of neurosurgery. 2018;1(aop):1–9. 10.3171/2018.2.JNS172327 30074456

[11] Bissonnette V, Mirchi N, Ledwos N, Alsidieri G, Winkler-Schwartz A, Del Maestro RF. Artificial Intelligence Distinguishes Surgical Training Levels in a Virtual Reality Spinal Task. JBJS. 2019;101(23):e127. 10.2106/JBJS.18.01197 PMC7406145 31800431

[12] Winkler-Schwartz A, Yilmaz R, Mirchi N, Bissonnette V, Ledwos N, Siyar S, et al Assessment of Machine Learning Identification of Surgical Operative Factors Associated With Surgical Expertise in Virtual Reality Simulation. JAMA Network Open. 2019;2(8):e198363–e198363. 10.1001/jamanetworkopen.2019.8363 31373651

[13] Delorme S, Laroche D, DiRaddo R, Del Maestro RF. NeuroTouch: A Physics-Based Virtual Simulator for Cranial Microneurosurgery Training. Operative Neurosurgery. 2012;71(suppl_1):ons32–ons42. 10.1227/NEU.0b013e318249c744 22233921

[14] Ruikar DD, Hegadi RS, Santosh K. A systematic review on orthopedic simulators for psycho-motor skill and surgical procedure training. Journal of medical systems. 2018;42(9):168 10.1007/s10916-018-1019-1 30073548

[15] Szasz P, Louridas M, Harris KA, Aggarwal R, Grantcharov TP. Assessing technical competence in surgical trainees: a systematic review. Annals of surgery. 2015;261(6):1046–55. 10.1097/SLA.0000000000000866 25119118

[16] Goff BA, Nielsen PE, Lentz GM, Chow GE, Chalmers RW, Fenner D, et al Surgical skills assessment: a blinded examination of obstetrics and gynecology residents. American journal of obstetrics and gynecology. 2002;186(4):613–7. 10.1067/mob.2002.122145 11967481

[17] Winkler-Schwartz A, Marwa I, Bajunaid K, Mullah M, Alotaibi FE, Bugdadi A, et al A comparison of visual rating scales and simulated virtual reality metrics in neurosurgical training: a generalizability theory study. World neurosurgery. 2019. 10.1016/j.wneu.2019.03.059 30880209

[18] Winkler-Schwartz A, Bissonnette V, Mirchi N, Ponnudurai N, Yilmaz R, Ledwos N, et al Artificial Intelligence in Medical Education: Best Practices Using Machine Learning to Assess Surgical Expertise in Virtual Reality Simulation. Journal of surgical education. 2019;76(6):1681–90. 10.1016/j.jsurg.2019.05.015 31202633

[19] Rosenblatt F. The perceptron: a probabilistic model for information storage and organization in the brain. Psychological review. 1958;65(6):386 10.1037/h0042519 13602029

[20] Wagner CR, Stylopoulos N, Jackson PG, Howe RD. The benefit of force feedback in surgery: Examination of blunt dissection. Presence: teleoperators and virtual environments. 2007;16(3):252–62.

[21] Sawaya R, Bugdadi A, Azarnoush H, Winkler-Schwartz A, Alotaibi FE, Bajunaid K, et al Virtual Reality Tumor Resection: The Force Pyramid Approach. Operative Neurosurgery. 2018;14(6):686–96. 10.1093/ons/opx189 . 28962033

[22] Ladha L, Deepa T. Feature selection methods and algorithms. International journal on computer science and engineering. 2011;3(5):1787–97.

[23] Li J, Cheng K, Wang S, Morstatter F, Trevino RP, Tang J, et al Feature selection: A data perspective. ACM Computing Surveys (CSUR). 2018;50(6):94.

[24] Yu L, Liu H. Efficient feature selection via analysis of relevance and redundancy. Journal of machine learning research. 2004;5(Oct):1205–24.

[25] Huan L, Lei Y. Toward integrating feature selection algorithms for classification and clustering. IEEE Transactions on Knowledge and Data Engineering. 2005;17(4):491–502. 10.1109/TKDE.2005.66

[26] Bottou L. Large-scale machine learning with stochastic gradient descent. Proceedings of COMPSTAT'2010: Springer; 2010. p. 177–86.

[27] Deo RC. Machine learning in medicine. Circulation. 2015;132(20):1920–30. 10.1161/CIRCULATIONAHA.115.001593 26572668 PMC5831252

[28] Jain A, Nandakumar K, Ross A. Score normalization in multimodal biometric systems. Pattern recognition. 2005;38(12):2270–85.

[29] Wang X, Wang Y, Wang L. Improving fuzzy c-means clustering based on feature-weight learning. Pattern recognition letters. 2004;25(10):1123–32.

[30] Ryu WHA, Chan S, Sutherland GR. Supplementary educational models in Canadian neurosurgery residency programs. Canadian Journal of Neurological Sciences. 2017;44(2):177–83. 10.1017/cjn.2016.315 27817764

[31] Sweller J. Cognitive load theory, learning difficulty, and instructional design. Learning and instruction. 1994;4(4):295–312.

[32] Gobet F, Lane PC, Croker S, Cheng PC, Jones G, Oliver I, et al Chunking mechanisms in human learning. Trends in cognitive sciences. 2001;5(6):236–43. 10.1016/s1364-6613(00)01662-4 11390294

[33] Block JH, Burns RB. 1: Mastery learning. Review of research in education. 1976;4(1):3–49.

[34] Williamson B. Digital education governance: data visualization, predictive analytics, and ‘real-time’policy instruments. Journal of Education Policy. 2016;31(2):123–41.

[35] Raskin JS, Liu JJ, Holste K, Brown S, Hardaway F, Pang P, et al Use of Risk Model for Assessment of Residents’ Perception of Complexity of Surgical Steps: Example of Modular Component Steps of Lumbar Spinal Fusion Surgery. Operative Neurosurgery. 2017;14(2):178–87. 10.1093/ons/opx072 29351677

[36] AlOtaibi F, Al Zhrani G, Bajunaid K, Winkler-Schwartz A, Azarnoush H, Mullah M, et al Assessing Neurosurgical Psychomotor Performance: Role of Virtual Reality Simulators, Current and Future Potential. SOJ Neurol. 2015;2(1):1–7.

[37] Carol-anne EM, Regehr G, Mylopoulos M, MacRae HM. Slowing down when you should: a new model of expert judgment. Academic Medicine. 2007;82(10):S109–S16. 17895673 10.1097/ACM.0b013e3181405a76

[38] Yuan Z-Y, Zhang D-Y, Yin Q, Liu Q, Shi D-C, Sun M-G, editors. Endoscopic Image Classification Based on DWT-CM and Improved BNN for Surgical Tool Appearances. 2007 International Conference on Machine Learning and Cybernetics; 2007: IEEE.

[39] Si W, Liao X, Wang Q, Heng P-A, editors. Augmented reality-based personalized virtual operative anatomy for neurosurgical guidance and training. 2018 IEEE Conference on Virtual Reality and 3D User Interfaces (VR); 2018: IEEE.

[40] Azarnoush H, Siar S, Sawaya R, Al Zhrani G, Winkler-Schwartz A, Alotaibi FE, et al The force pyramid: a spatial analysis of force application during virtual reality brain tumor resection. Journal of neurosurgery. 2016;127(1):171–81. 10.3171/2016.7.JNS16322 27689458

[41] Norman D. The design of everyday things: Revised and expanded edition: Constellation; 2013.

[42] Calvo RA, Peters D. Positive computing: technology for wellbeing and human potential: MIT Press; 2014.

[43] Ende J. Feedback in clinical medical education. Jama. 1983;250(6):777–81. 6876333

[44] Hajshirmohammadi I. Using fuzzy set theory to objectively evaluate performance on minimally invasive surgical simulators: School of Engineering Science-Simon Fraser University; 2006.

[45] Burbidge R, Trotter M, Buxton B, Holden S. Drug design by machine learning: support vector machines for pharmaceutical data analysis. Computers & chemistry. 2001;26(1):5–14. 11765851 10.1016/s0097-8485(01)00094-8

[46] Ten Cate O, Snell L, Mann K, Vermunt J. Orienting teaching toward the learning process. Academic Medicine. 2004;79(3):219–28. 10.1097/00001888-200403000-00005 14985194

