# Supplementary material for: The Virtual Operative Assistant: An explainable artificial intelligence tool for simulation-based training in surgery and medicine
Source: PLoS One. 2020 Feb 27;15(2):e0229596. doi: 10.1371/journal.pone.0229596 (PMC7046231; doi:10.1371/journal.pone.0229596)
Supplement: S1 File — (DOCX) [file pone.0229596.s001.docx]

**Supplementary material**

**Table of contents**

S1 Appendix. Rationale for machine learning-based framework 2

S2 Appendix. Individual metric scoring cases 5

References 8

**S1 Appendix. Rationale for machine learning-based framework**

Supervised algorithms are a subset which involve labelling training data, in order to allow the algorithm to recognize hidden patterns in the data and make predictions regarding the class of new, unlabelled data.[1] In the training process, a set of input data called features (designated as ***x***) and output class (designated as ***Y***) are supplied to the algorithm.[1] The system then proceeds to create a hypothesis (***h***). A hypothesis is a function created by the learning algorithm based on the training data (S3 Figure).

During training, the hypothesis, ***h(x),*** attempts to assign a weight (**𝜽**) to each input (***x***), to output a predicted ***Y*** (equation 1 and equation 2).[1] The first weight (**𝜽_0_**) does not have an assigned metric and therefore corresponds to the bias which we observe in the perceptron.[1] The input (***x***) are a column vector and ***T*** denotes the transpose.

**Equation 1**

$$h\left( x \right)= \theta_{0} +\theta_{1}x_{1} + \theta_{2}x_{2} + ... + \theta_{n}x_{n}$$

**Equation 2**

$$h\left( x \right)=\left[ \theta_{0} \theta_{1} \theta_{2} \ldots\theta_{n} \right]\left[ \begin{aligned} x_{0} \\ x_{1} \\ x_{2} \\ . \\ . \\ . \\ x_{n} \end{aligned} \right]= \theta^{T}x$$

For each iteration, the predicted ***Y*** is compared to the real class, thereby providing insight on the performance of the hypothesis. A common approach to measure the performance of the hypothesis, is to calculate the cost.[2] If the cost of a hypothesis is large, the hypothesis may be able to improve by modifying its weights. Several optimization methods as well as more simple gradient descent techniques exist to provide a standardized method to reduce cost.[2] Following several iterations, the cost will reach a minimum, whereby the performance of the hypothesis has been optimized.[2]

Once a hypothesis has been optimized, it can be referred to as a model. For illustration purposes, the model can be represented as a logistic sigmoid function (equations 3, 4, and 5).

**Equation 3**

$$h\left( x \right)= g\left( \theta^{T}x \right)$$

**Equation 4**

$$z= \theta^{T}x$$

**Equation 5**

$$g\left( z \right)= \frac{1}{1+ e^{-z}}$$

In this fashion, this algorithm’s decision boundaries can be more easily visualized.[2] The output of the hypothesis, ***h(x)***, represent the probability that ***Y*** = 1 for a given set of inputs ***x*** with weights **𝜽**.[2] The algorithm is now represented by ***z***, and ***g(z)*** is analogous to ***h(x)***. S4 Figure illustrates a plot whereby the x-axis represents the output of the hypothesis function, and the y-axis is the probability that ***Y*** = 1.

Following the figure above, it is clear that when the model yields a positive value for ***z***, the probability that ***Y*** = 1 is above 0.5. Hence the prediction will be +1. However, if the output of the model is negative, the probability that ***Y*** = 1 is below 0.5. The prediction would therefore be -1 in this case. Interestingly, we can notice that as the model yields an increasingly positive value for ***z***, the probability of ***Y*** = 1 approaches 1. This principle is of particular importance to the Virtual Operative Assistant as the user attempts to increase the value of their ***z*** to increase the probability of being correctly classified as an expert. The Virtual Operative Assistant also takes advantage of this by providing a probability for each of the two classes (skilled and novice).

Once the decision boundaries have been elucidated, we can begin to understand the impact of individual weights, **𝜽**, on an algorithm’s decision-making. The weights may be negative or positive. The larger the absolute value of a weight, the larger the influence of it corresponding input ***x***.[3] For example, in the context of surgical expertise, if the skilled class is represented by ***Y*** = +1 and novice as ***Y*** = -1, a small increase in a metric with a large positive weight will cause a larger shift towards the positive output than one with a small weight. In addition, if a weight is negative, a skilled surgeon is expected to achieve a larger negative metric in order to maximize the positive output.

**S2 Appendix. Individual metric scoring cases**

In the case of surgical simulation, four possible cases exist below. For simplicity purposes, the example are shown for 3 metrics, and the biases are left out.

**Case 1.** The output (***h(x)***) is positive, corresponding to a skilled user. The user is also considered a skilled in each individual metric (positive value for **𝜃 * *x***). An example of this scenario with three metrics following equation 1 from the S1 Appendix would be as follows:

**Equation 6**

$$h\left( x \right)=\left( 0\text{.}5*1 \right)+\left( -1\text{.}2*-0\text{.}3 \right)+(1\text{.}4*2\text{.}1)$$

In this case, the user is scoring positively in all the assessed metrics, thereby indicating expertise in all components of the model’s decision.

**Case 2.** The output (***h(x)***) is positive, corresponding to a skilled user. However, the user is only considered skilled in some, but not all, of the metrics assessed. Skilled metrics (positive value for **𝜃 * *x***) therefore overcompensate for novice metrics (negative value for **𝜃 * *x***), thereby allowing for an overall positive output. An example of this scenario with three metrics following equation 1 would be as follows:

**Equation 7**

$$h\left( x \right)=\left( 0\text{.}5*1 \right)+\left( -1\text{.}2*0\text{.}3 \right)+(1\text{.}4*2\text{.}1)$$

In this case, the user is scoring positively in the first and last metric. However, the user scores negatively in metric 2. This indicates that the user may still improve performance for metric 2. Nonetheless, as the sum of the product of each metric and their weight is positive, this user is classified as skilled.

**Case 3.** The output (***h(x)***) is negative, corresponding to a novice user. However, the user is only considered novice in some, but not all, of the metrics assessed. The novice metrics (negative value for **𝜃 * *x***) overcompensate for this individual’s skilled metrics (positive value for **𝜃 * *x***), thereby resulting in an overall negative output. An example of this scenario with three metrics following equation 1 would be as follows:

**Equation 8**

$$h\left( x \right)=\left( 0\text{.}5* -1 \right)+\left( -1\text{.}2*0\text{.}3 \right)+(1\text{.}4*2\text{.}1)$$

In this case, the user is scoring negatively in the first and second metric, however the user scores positively in metric 3. This indicates that the user should focus on improving performance in the first two metrics, whereas metric 3 performance is within the skilled range.

**Case 4.** The output (***h(x)***) is negative, corresponding to a novice user. The user is also considered novice in each individual metric (negative value for **𝜃 * *x***). An example of this scenario with three metrics following equation 1 would be as follows:

**Equation 9**

$$h\left( x \right)=\left( 0\text{.}5*-1 \right)+\left( -1\text{.}2*0\text{.}3 \right)+(1\text{.}4* -2\text{.}1)$$

In this case, the user is scoring negatively in all of the metrics. Hence, the user needs to improve performance in all metrics in order to increase their probability of being classified as skilled.

**Supplementary references**

1. Alpaydin E. Introduction to machine learning: MIT press; 2009.

2. Bishop CM. Pattern recognition and machine learning. New York: Springer; 2006.

3. Wang X, Wang Y, Wang L. Improving fuzzy c-means clustering based on feature-weight learning. Pattern recognition letters. 2004;25(10):1123-32.
