# Supplementary figures and images for: The Virtual Operative Assistant: An explainable artificial intelligence tool for simulation-based training in surgery and medicine
Source: PLoS One. 2020 Feb 27;15(2):e0229596. doi: 10.1371/journal.pone.0229596 (PMC7046231; doi:10.1371/journal.pone.0229596)

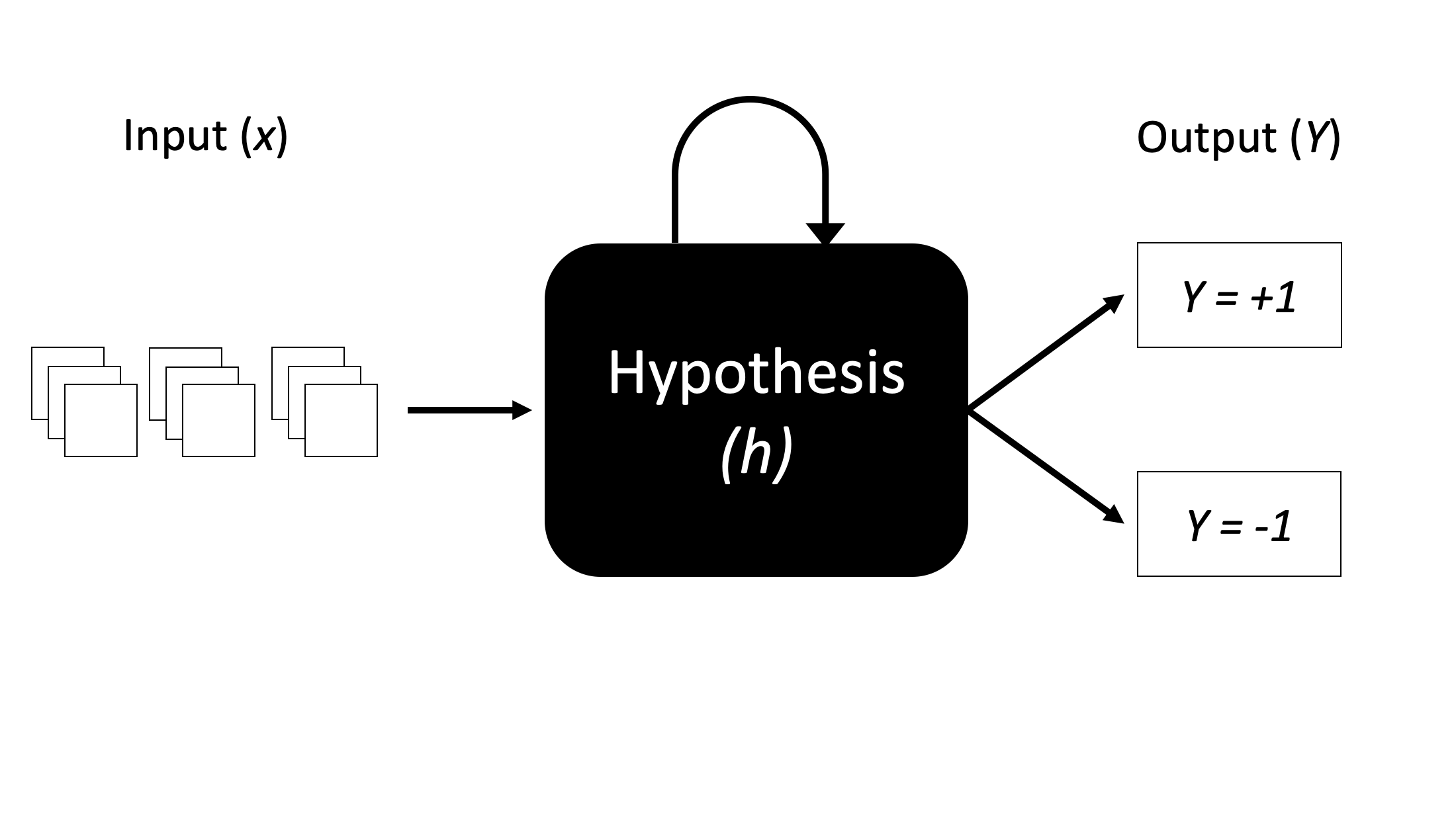

Supplement: S1 Fig — (TIFF) [file pone.0229596.s002.tiff]

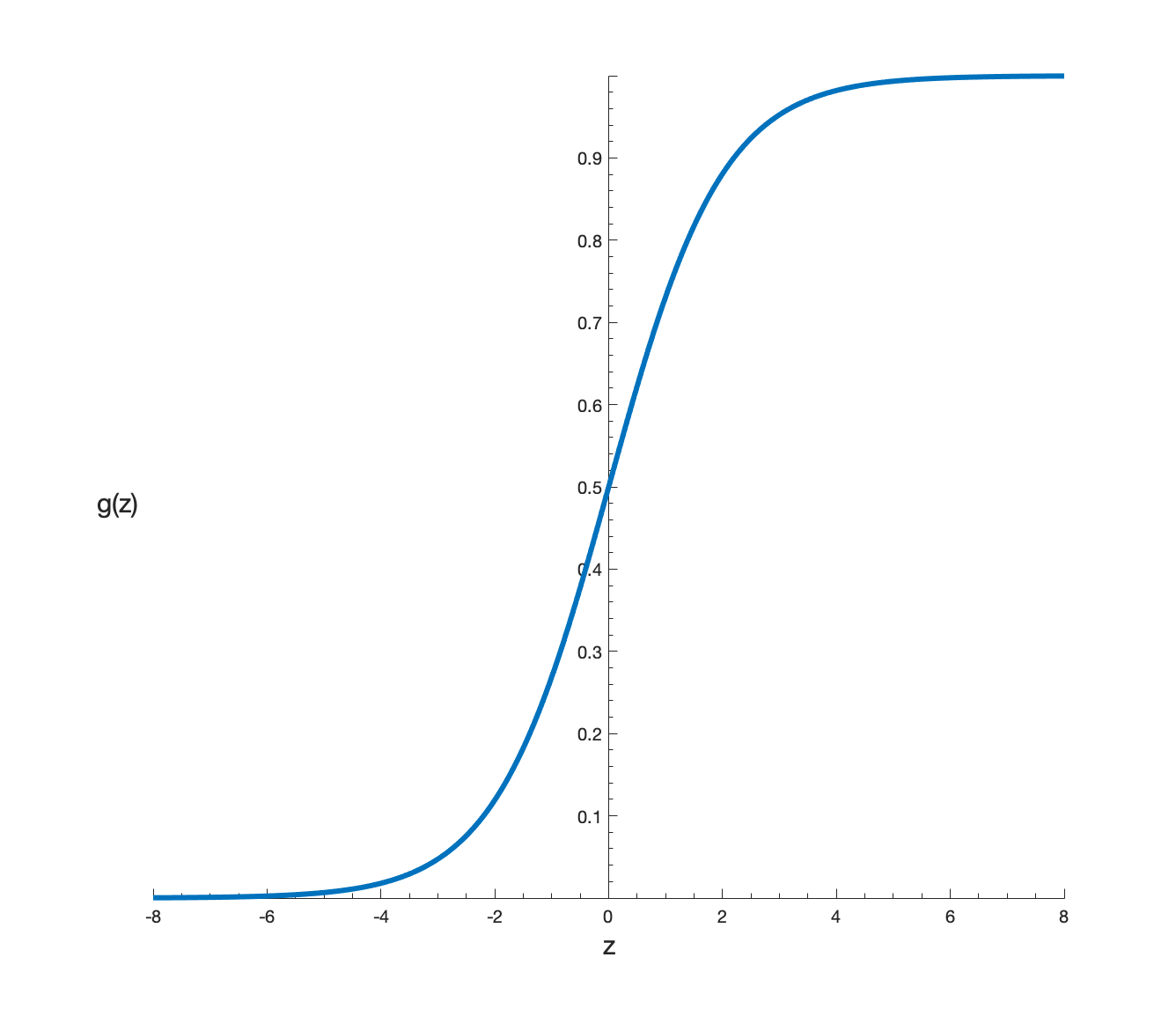

Supplement: S2 Fig — (TIF) [file pone.0229596.s003.tif]
